# The Perfect Timing—Immediate versus Delayed Microvascular Reconstruction of the Mandible

**DOI:** 10.3390/cancers16050974

**Published:** 2024-02-28

**Authors:** Daniel G. E. Thiem, Fabia Siegberg, Shankeeth Vinayahalingam, Sebastian Blatt, Maximilian Krüger, Bernd Lethaus, Bilal Al-Nawas, Rüdiger Zimmerer, Peer W. Kämmerer

**Affiliations:** 1 Department of Oral and Maxillofacial Surgery, Facial Plastic Surgery, University Medical Centre Mainz, Augustusplatz 2, 55131 Mainz, Germany; siegberf@uni-mainz.de (F.S.); blattseb@uni-mainz.de (S.B.); maximilian.krueger@unimedizin-mainz.de (M.K.); al-nawas@uni-mainz.de (B.A.-N.); peer.kaemmerer@unimedizin-mainz.de (P.W.K.); 2 Department of Oral and Maxillofacial Surgery, Radboud University Medical Center, 6525 GA Nijmegen, The Netherlands; shankeeth.vinayahalingam@radboudumc.nl; 3 Department of Oral and Maxillofacial Surgery, Facial Plastic Surgery, University Medical Centre Tübingen, Osianderstr. 2-8, 72076 Tübingen, Germany; bernd.lethaus@med.uni-tuebingen.de (B.L.); ruediger.zimmerer@web.de (R.Z.); 4 Department of Oral and Maxillofacial Surgery, Facial Plastic Surgery, University Medical Centre Leipzig, Liebigstraße 12, 04103 Leipzig, Germany

**Keywords:** mandibular reconstruction, reconstruction timing, free flap, head and neck, economics

## Abstract

**Simple Summary:**

This study evaluated the clinical and economic impacts of immediate versus delayed reconstruction of the mandible in patients following resection. Data from two German oral and maxillofacial surgery university departments provided insights into patient demographics, surgical details, medical histories, and flap survival rates. Our analysis of 177 reconstructions (72 immediate, 105 delayed) revealed no significant difference in flap survival based on timing or radiotherapy. However, immediate reconstruction is less cost-intensive. The findings aim to inform surgical decision-making, balancing patient health and cost-effectiveness. Crucially, only 18% of patients achieved functional masticatory rehabilitation, underscoring the need for improved post-surgical care pathways.

**Abstract:**

In this retrospective study, the clinical and economic implications of microvascular reconstruction of the mandible were assessed, comparing immediate versus delayed surgical approaches. Utilizing data from two German university departments for oral and maxillofacial surgery, the study included patients who underwent mandibular reconstruction following continuity resection. The data assessed included demographic information, reconstruction details, medical history, dental rehabilitation status, and flap survival rates. In total, 177 cases (131 male and 46 females; mean age: 59 years) of bony free flap reconstruction (72 immediate and 105 delayed) were included. Most patients received adjuvant treatment (81% with radiotherapy and 51% combined radiochemotherapy), primarily for tumor resection. Flap survival was not significantly influenced by the timing of reconstruction, radiotherapy status, or the mean interval (14.5 months) between resection and reconstruction. However, immediate reconstruction had consumed significantly fewer resources. The rate of implant-supported masticatory rehabilitation was only 18% overall. This study suggests that immediate jaw reconstruction is economically advantageous without impacting flap survival rates. It emphasizes patient welfare as paramount over financial aspects in clinical decisions. Furthermore, this study highlights the need for improved pathways for masticatory rehabilitation, as evidenced by only 18% of patients with implant-supported dentures, to enhance quality of life and social integration.

## 1. Introduction

**Figure 1:**
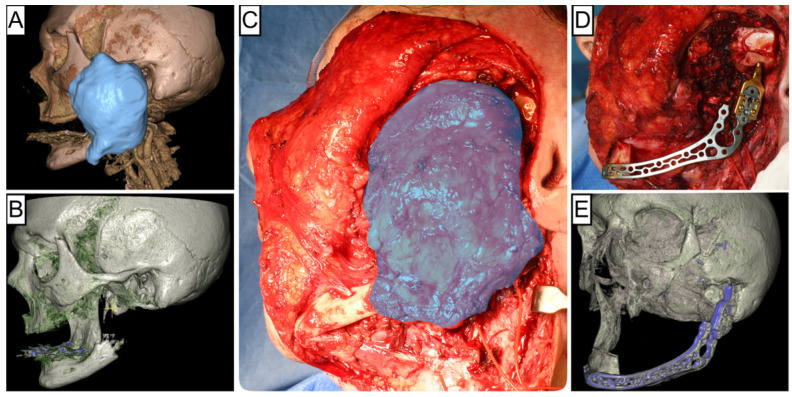
Illustration of an extended osteosarcoma located in the left ascending mandible. Preoperative 3D CT reconstruction highlighting the tumor volumetry in blue (**A**), 3D reconstruction showing the affected mandibular branch (**B**), clinical intraoperative view with the tumor outlined in blue (**C**), post-resection intraoperative view following alloplastic reconstruction (**D**), and postoperative 3D CT reconstruction depicting the surgical result (**E**).

**Figure 2:**
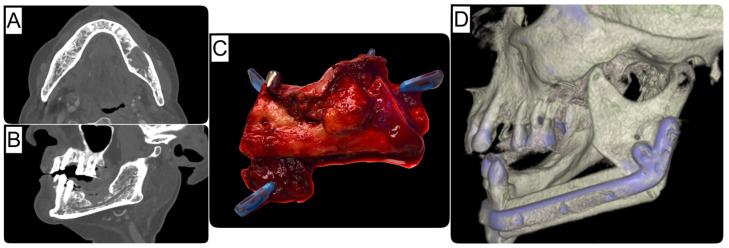
Case of an oral squamous cell carcinoma in the left mandible, with osseous infiltration. (**A**) Axial and (**B**) sagittal views from preoperative CT imaging. (**C**) Resected tumor specimen. (**D**) Postoperative situation after autologous and alloplastic (patient-specific implant) reconstruction as 3D image.

In reconstructive oral and maxillofacial surgery, free flap transfer represents one of the most important and frequently performed methods for defect reconstruction of the head and neck region. Flap survival is the primary criterion for success after free flap transfer and is generally considered very good at approximately 96% [1,2]. Partial or even complete flap loss significantly increases the morbidity of patients, prolongs hospital stays, and increases the cost to the health care system [3]. Large tumors involving the bone often require soft tissue and bony resection, resulting in extensive and, thus, functionally limiting hard and soft tissue defects [4]. For many years, there has been an ongoing debate about the appropriate timing for bony jaw reconstruction after tumor-ablative resection. The current treatment regimens can be categorized into immediate (one-step) and staged/delayed (two-step) bone reconstruction, depending on the local site situation, the patient’s state of general health and overall prognosis, the quality of the donor site, and the experience of the surgeon. In this context, maxillary and mandibular reconstruction should be fundamentally differentiated, as functional rehabilitation of the mandible can be primarily restored using a bridging osteosynthesis and a soft tissue flap [5,6]. Historically, proponents of a delayed or staged approach advocated a period of observation to monitor the patient for the development of recurrent disease or to establish histologically clear bony margins before reconstruction. Of course, there are cases in which the probability of an R0 resection for extensive tumors already appears questionable and uncertain preoperatively (Figure 1). In these cases, a two-stage approach for bony reconstruction is without question indicated and sensible. In cases with clinically and radiologically circumscribed tumor extension (Figure 2), the immediate approach is a proven treatment regime, especially since there is insufficient evidence or representative data allowing for a definition for the extent of a clinical bony safety margin. Further, it is widely accepted that immediate reconstruction may be performed without the risk of a worse prognosis [7,8]. Immediate reconstruction has several advantages over delayed reconstruction for mandibular reconstruction, including significantly fewer (50%) events of plate-related complications [9]. Furthermore, Boyd et al. reported that patients who underwent reconstruction with vascularized bone flaps experienced an average of four days of life lost for delayed procedures, compared to 35 days for patients who underwent alloplastic reconstruction with soft tissue flaps [10]. Health-related quality of life (QOL) studies are inconsistent regarding the timing of reconstruction, showing both significant [11,12] and no improvement in QOL following bony reconstruction [13]. A substantial reason for this is the heterogeneous rate of implant-supported oral rehabilitations. Therefore, this study aimed to compare immediate versus delayed microvascular reconstruction of the mandible after ablative surgery in terms of clinical and economic outcomes.

## 2. Materials and Methods

This retrospective study obtained patient data from the oral and maxillofacial surgery departments at the University Hospitals of Mainz and Leipzig. The analysis included all patients who underwent immediate or delayed mandibular reconstruction between 2009 and June 2022 and between 2014 and 2023, respectively. Reconstruction refers specifically to the bony restoration following continuity resection. Immediate reconstruction was characterized as restoring bony continuity concurrently with continuity resection.

Relevant cases were identified using the hospital’s internal 2016 SAP^®^ patient management software (Walldorf, Germany). The case search and selection were based on the International Statistical Classification of Diseases and Related Health Problems (ICD-10), German modification (ICD-10-GM), and the German surgical procedure classification (OPS 2021). The ICD-10-GM codes used were C06.-: malignant neoplasm of other and unspecified parts of the mouth and further subdivision thereof (C06.0, C06.1, C06.2); C02.-: malignant neoplasm of other and unspecified parts of the tongue and further subdivision thereof (C02. 0, C02.1, C02.2, C02.3, C02.9); C03.-: malignant subdivision of gum and further subdivision (C03.0, C03.1, C03.9); and C41.-: malignant neoplasm of bone and articular cartilage of other and unspecified locations and subdivision (C41.0, C41.1). The searched OPS codes are shown in the Supplementary Materials.

The data collected comprised patient demographics (age at reconstruction and gender), timing of reconstruction, free flap type, history of external radiotherapy (XRT) and chemotherapy, local recurrence status, diagnosis leading to reconstruction, reconstruction site, flap survival rate, time until reconstruction, implant-supported rehabilitation status, and time until implant-supported dental rehabilitation.

### Statistics

The raw data sets were saved in Excel^®^ 2021 sheets (Microsoft Corporation, Redmond, WA, USA) and transferred into SPSS Statistics^®^ (version 27., MacOS X; SPSS Inc., IBM Corporation, Armonk, NY, USA). The data are expressed as mean, standard deviation (±SD), minimum (min), and maximum (max). Correlation analyses were calculated using the Pearson Chi-Square test for expected frequencies ≥ 5. The binomial test was used to test whether the frequency distribution of a dichotomous variable corresponds to a presumed distribution. *p*-values ≤ 0.05 were termed significant. Line and bar charts were used for illustration purposes.

## 3. Results

A summary of the patient characteristics can be found in Table 1. This retrospective study gathered data from two German universities’ oral and maxillofacial surgery departments (University A: January 2010–January 2023; University B: January 2014–November 2022). A total of 177 cases with bony free flap mandibular reconstruction were included, with 72 immediate and 105 delayed reconstruction cases. The cohort consisted of 131 males and 46 females. The mean age for immediate reconstruction was 59 years (range: 14–83 years), and for delayed reconstruction, it was 59 years (range: 18–87 years). Adjuvant radiotherapy (XRT) was administered to 144 out of 177 patients (81%), while combined radio- and chemotherapy were provided to 91 out of 177 patients (51%). When stratified by gender, there were no significant differences for surgical indication, donor tissue used for the reconstruction, cause of flap failure, or radiation history. Reconstruction following radiotherapy at the recipient site, including the recipient vessel area, was performed in 93 cases—4 immediate and 89 delayed reconstructions (Table 1).

Malignant tumor resection was the primary surgical indication (75.1%, n = 133/177), followed by continuity resection due to osteoradionecrosis (ORN; 10.7%, n = 19/177) (Table 2).

Out of 177 flaps, 29 were lost, yielding a flap loss rate of 16.4%. No significant differences in total case count were observed between the two departments (Table 3).

**Figure 3:**
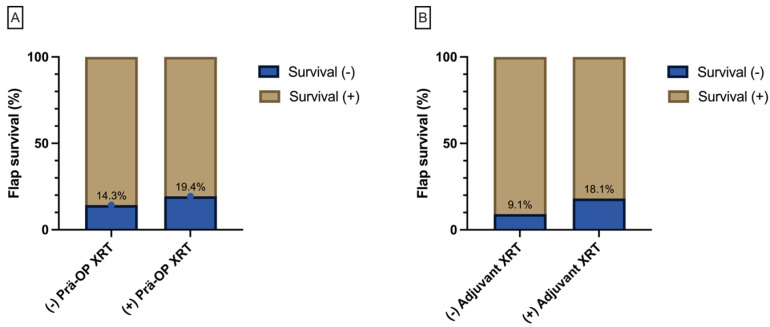
The bar graph displays the percentage of free flap survival for preoperative radiotherapy (**A**) and its correlation with the status of adjuvant radiotherapy (**B**).

Regarding reconstruction timing, 37.9% (11/29) of flap losses occurred after immediate reconstruction, while 62.1% (18/29) followed delayed reconstruction; however, no significant association between reconstruction timing and flap survival was detected, (Chi-Square(1) = 0.11, *p* = 0.74) with 11 flap losses out of the 77 immediate and 18 out of the 105 delayed reconstructions. Flap survival was not correlated with either preoperative radiotherapy (Chi-Square (1) = 1.23, *p* = 0.26; Figure 3) or adjuvant (postoperative) radiotherapy (Chi-Square(1) = 1.57, *p* = 0.21) (Figure 3).

The mean time until reconstruction was 14.5 months (min: 0; max: 196). When studying the relationship between flap loss and the period from resection to reconstruction, logistic regression revealed a regression coefficient (B) of 0.006, accompanied by a standard error of 0.010. The calculated p-value for this variable was 0.507, which exceeds the conventionally accepted significance threshold of 0.05. Consequently, the data do not provide sufficient evidence to support the hypothesis that the duration preceding reconstructive surgery significantly influences the free flap loss rate. In conclusion, the current model does not establish a robust correlation between free flap loss and the time interval before reconstruction. Dental rehabilitation status information was available for 102 of the 177 patients. Among them, 7% received implant-supported prosthetic rehabilitation following immediate bone reconstruction, and 29% after delayed reconstruction, amounting to a total rehabilitation rate of 18%.

### Two-Department Economic Analysis of Mandibular Reconstructions from 2010 to 2023

**Figure 4:**
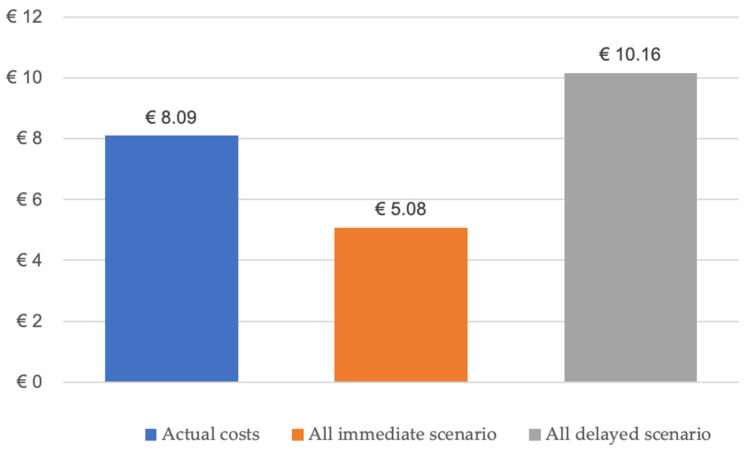
Bar chart shows the average case revenue depending on the procedure performed and the reconstruction time.

From 2010 to 2023, mandibular reconstruction following tumor resection was performed on 177 patients. Immediate reconstructions were conducted in 72 cases, while 105 were delayed reconstructions. The accumulated revenue for the immediate reconstructions during this span would have amounted to EUR 2,066,784.48, taking an average income per case of EUR 28,705.34 (mean annual base case value from 2010 to 2023), presenting the appropriate categorization of DRG-D02A, denoting complex head and neck resections with intricate or combined procedures with extremely severe CC. To compare the treatment costs of different therapeutic regimens, it is essential to account for the sum of expenses/revenues from the procedure with continuity resection and the isolated delayed reconstruction (bone reconstruction). Hence, during the specified period, the total invoiced payment for the delayed reconstruction amounted to EUR 6,028,121.40. This would result in an estimated amount for immediate and delayed reconstructions of EUR 8,094,905.88. Had all 177 reconstructions been executed as immediate procedures (EUR 5,080,845.18), there would have been a revenue decrease for the clinics or a health expenditure reduction of EUR 3,014,060.70 (−37%). In comparison, had all reconstructions been executed as delayed procedures (EUR 10,161,690.36), the clinics’ revenue would have been increased by EUR 2,066,784.48 (Figure 4).

## 4. Discussion

In this retrospective analysis, we assessed the influence of reconstruction timing on clinical and economic perspectives after microvascular reconstruction of the mandible in patients from two German university departments. The two studied cohorts exhibited an almost identical average age and a frequency distribution favoring male patients. In both groups, OSCC was the most common cause for mandibular reconstruction, accounting for 79% and 76%, respectively. The department-independent analysis revealed no significant correlation between the timing of reconstruction and flap survival (χ^2^ = 0.74), consistent with the prevailing literature [14]. In this context, it should be noted that the comparison of the two groups is subject to a certain degree of inaccuracy due to the different sample sizes. Group A consisted of 72 people, while Group B consisted of 105 people. This discrepancy between group sizes may lead to a potential bias or affect the statistical power of our analyses. It is also essential to highlight the differing procedural preferences between the departments. Department A exhibited a relatively balanced distribution with 55% immediate and 45% delayed reconstructions. In contrast, Department B opted for immediate reconstruction in only 24% of cases, with delayed reconstruction accounting for the remaining 76%. In addition, Department A, when opting for the less frequently performed delayed approach, showed an 18% flap loss compared to 11% in the primary approach. Despite more frequent delayed reconstructions, Department B had a lower (16%) flap loss compared to 26% with the immediate approach, underscoring the importance of procedural routine [15]. Within both departments, there is no dogmatic stance or preference towards either immediate or delayed reconstruction timing. The decision for immediate versus delayed reconstruction is consistently based on the individual’s specific local findings, the expected probability of a margin-free (R0) resection, as well as the patient’s preferences and overall health condition. In cases where there is already a disproportionately high anesthesia risk due to extensive cardiovascular comorbidities, primary reconstruction is always preferred to avoid a secondary procedure. Essentially, the experience and individual assessments of the treating surgeons also play a significant, though unquantifiable, role. Therefore, there is no rigid algorithm for deciding between an immediate or delayed approach in the decision-making process. However, mandibular reconstruction remains a significant challenge for surgical teams globally, and the literature currently lacks comprehensive data on the optimal timing for reconstruction, indicating no established clinical standard in both the European and Anglo-American regions [16]. Arguments for the delayed approach cite concerns over local recurrence monitoring, prolonged primary surgery leading to increased morbidity from cardiopulmonary issues, and delayed adjuvant radiotherapy (XRT) onset. Although the assumption of an increased local recurrence rate in the context of immediate reconstruction has not yet been confirmed with final evidence, the first studies were able to reject a positive correlation [7]. In this study, the local recurrence rates were 21.1% for the immediate bony reconstruction group and 26.7% within the group who received delayed bony reconstruction. Both values are consistent with those in the relevant literature, which showed local recurrence rates between 20% and 30% in patients receiving a two-step approach [17] and those with immediate reconstruction (22.6%) [18]. In terms of the duration of surgery, there is solid evidence indicating a correlation between the length of the surgery and the risk of flap failure [19]. A study using a national inpatient database from Japan examined risk factors for free flap failure in patients with head and neck cancers. They found that a duration of anesthesia >18 h was associated with a higher risk of flap failure [20]. Wong et al. also used a multi-institutional database to demonstrate that patients undergoing a surgery with a duration equal to or greater than the 75th percentile (625.5 min) were twice as likely to result in flap failure [21]. In this context, however, attention must be paid to the heterogeneity of the study data and the providing institutions. In many specialized departments, the two-team approach is now used by default to minimize surgical time. Similar conclusions were reached by Offodile et al. as they demonstrated a significant increase in the likelihood of early flap failure with prolonged surgical time (6–12 h: OR: 4.28 and >12 h: OR: 6.41, *p* < 0.0001) based on 2008 patients from the ACS-NSQIP database [22]. For shorter surgery durations (<625.5 min), however, no difference between immediate and delayed bony reconstructions is evident [23]. A reduction in surgical time as a reason for the two-stage approach in reconstruction must be critically analyzed since most cases of delayed reconstruction also involve using a free flap (even if only a soft-tissue flap is used) for defect reconstruction or plate coverage. In this context, it was demonstrated that the duration of flap elevation only differs slightly (3.8–27.7 min) in the two frequently used free flaps (radial free forearm flap and free fibular flap) [24]. The site of reconstruction also seems to significantly influence flap survival. Next to the restoration of function and aesthetics, sufficient flap perfusion is the main priority during the first few postoperative days. Most flap failures occur within the first postoperative 72 h, which are classified as early flap failures [25,26]. Several factors have been identified in the medical literature that correlate with free flap failure, including intraoperative and postoperative fluid management, diabetes mellitus [27], prior radiation or chemotherapy, patient age, tobacco and alcohol use, postoperative alcohol withdrawal, Body Mass Index (BMI), and surgical duration [28,29,30]. Notably, though infrequently delineated, extended ischemic time was discovered to significantly impact flap viability. Moreover, intraoperative pedicle revision was considered an independent risk determinant for free flap failure [30].

Proponents of delayed bony reconstruction report higher flap failure rates due to adjuvant radiotherapy following cancer resection. Although the literature is inconsistent, different studies have failed to show a significant impact of direct adjuvant radiation exposure on flap survival [31,32]. No correlation could be identified in the present work either. However, an increased risk of complications in previously irradiated patients is widely acknowledged, showing that patients with previous XRT were at an increased risk of total flap failure [33], partial flap failure, and overall postoperative complications including local infection, plate exposure, and wound dehiscence [31,34,35,36]. Regarding the occurrence of malperfusion, there is a positive correlation with reconstruction timing, as delayed procedures (>15 weeks after XRT) are associated with a higher rate of vascular complications [36]. Percutaneous irradiation of the cervical lymphatic drainage region is assumed to alter the recipient vessels’ architecture, including intimal proliferation, hyaline thickening, and thrombosis [37,38]. In addition, findings of advanced atherosclerosis and fibrosis after fractionated irradiation and electron microscopic evidence of dehiscence of the intima may explain an excessive activation of the coagulation cascade with subsequent vascular pedicle thrombosis [39]. The present study found no correlation between flap failure rate and adjuvant XRT status. However, this could be related to the number of patients or the high expertise in microvascular reconstruction in both departments.

In this context, the rate of implant-supported prosthetic rehabilitation was significantly higher at 29% after delayed reconstruction compared to immediate reconstruction with 7%. This discrepancy could be attributed to a more pronounced desire among patients for dental rehabilitation, often serving as a driving factor in the decision-making process for mandibular reconstruction. Regarding the support of dental rehabilitation by the national health system in Germany, the distinction between the private health insurance system and the compulsory state insurance system plays a pivotal role in access to and funding for implant-supported dentures. Private health insurance typically covers the costs of such treatments in most scenarios, offering a contrast to the basic statutory health insurance, which does not routinely include this form of treatment. However, statutory insurance may cover the costs under exceptional circumstances, particularly in cases of malignancy-related tooth loss or when such rehabilitation is necessitated by the outcomes of cancer treatments like radiotherapy or surgery. This delineation within the insurance system introduces a complex, often bureaucratic process for approval and coverage, necessitating substantial involvement from treating physicians to support their patients’ cases. The time-consuming nature of this process, coupled with its bureaucratic demands, results in significant delays—frequently up to a year between application and treatment. During this interim, patients may experience deterioration in their ability to chew and swallow, a reduction in dietary variety, and a decrease in mouth opening due to scar formation, all of which are exacerbated by the absence of adequate dental prosthetics. These factors collectively contribute to a landscape where the provision of timely and effective dental rehabilitation post-mandibular reconstruction is fraught with challenges. The implications of this situation on our study’s conclusions are profound, suggesting that while our findings provide valuable insights, they must be interpreted within the context of these systemic healthcare limitations. In a hypothetical scenario in which the costs of implant-supported masticatory rehabilitation are covered by health insurance, the simultaneous insertion of endosseous implants during osseous reconstruction, which in the age of computer-aided reconstruction planning in the sense of backward planning would be entirely possible, would be one way of addressing this deficiency. At the same time, this approach would be associated with possible damage to the transplanted and highly fragile tissue, which represents a risk factor for the success of the reconstruction and is therefore avoided by most surgeons. While the delayed approach described above has already been carried out and is undoubtedly predictable, the adjuvant radiotherapy regularly required after primary reconstruction represents an influencing factor that compromises the necessary osseointegration, which severely limits the predictability of successful treatment. In order to improve the care situation of affected patients for the benefit of all parties involved, i.e., the patients at the center, their practitioners, and the health insurance companies processing the treatment, and thus reduce the bureaucratic burden, it would make sense to integrate the costs for implant-supported dentures into the treatment plan and thus the overall reimbursement. It would be conceivable to define sufficient graft healing and completed adjuvant radiotherapy as a prerequisite, so that the risk of implant loss is reduced, taking into account the already suboptimal soft tissue situation. 

In Germany, the most frequently triggered DRG case rate in this context is D02A, which pertains to complex resections and reconstructions in the head and neck area with intricate or combined procedures involving severe CC. Between 2010 and 2021, the DRG-D02A, associated with ICD-10 codes related to the lower jaw (13,965 instances) and upper jaw (15,959 instances), was billed. This indicates an estimated 87.5% involvement of the lower jaw. During the same period, the OPS codes 5-858.80 (transplantation of an osteomyocutaneous or osteofasciocutaneous flap to the head and neck) and 5-77b.4 (vascularized bone grafting with microvascular anastomosis) were coded 2274 times. With 87.5% mandibular involvement and 50% delayed reconstructions (a hypothetical value due to the lack of a registry for Germany), approximately 995 cases would have involved delayed reconstructions of the lower jaw from 2010 to 2021. Due to the procedure’s complexity, delayed jaw reconstruction in tumor patients must be coded as DRG case rate D02A. With an average revenue of EUR 28,303.74 per case from 2010 to 2021, the costs for this period would be EUR 56,324,442.60.5.

### Limitations

The topic addressed in this study focuses on a specialized treatment method for a diverse patient group. This reconstruction procedure is primarily used in the surgical treatment of patients with head and neck tumors, which rank eighth among the most common solid cancers worldwide. However, a significantly smaller proportion of these patients undergo mandibular continuity resection, diminishing its quantitative significance in annual healthcare expenditures compared to more prevalent cancers like prostate and breast carcinoma. While the bicentric approach enhances the representativeness of the results, the total of 177 cases (72 for primary reconstruction vs. 105 for delayed reconstruction) allows for only a limited conclusive assessment. One of the acknowledged limitations of this study is the heterogeneity of the lesions examined, which span a spectrum from benign to malignant, with some represented in relatively low numbers. This diversity, while reflective of the real-world clinical setting, introduces complexity in interpreting the study’s findings and their applicability across different lesion types. The variance in lesion characteristics and their respective frequencies may impact the robustness and generalizability of our conclusions. Moreover, the limited representation of certain lesion types could affect the statistical power of our analyses concerning those specific categories. Additionally, the reported number for immediate and delayed reconstructions nationwide should be interpreted cautiously due to the absence of comprehensive statistics. The missing data on dental rehabilitation status indeed mirror a broader, systemic issue within the healthcare landscape, specifically the under-supply of tumor patients after jaw resection with implant-supported dentures. This gap in data collection and documentation reflects not just a limitation of our study but also a critical area of need within patient care pathways post-reconstruction. The incomplete data presented in our study, while aligned with the existing literature, indeed holds the potential to skew our findings. It is plausible that undocumented cases might have received implant-retained prostheses, which could significantly alter the interpretation of our results towards a more favorable situation after immediate reconstructions and in total. This possibility underlines the need for continuous and comprehensive data documentation in everyday clinical practice and the importance of considering the broader health context in which these treatments take place.

## 5. Conclusions

Microvascular reconstruction of the mandible is a well-established therapeutic procedure, though its optimal timing remains a subject for ongoing debate. The decision is influenced by individual patient characteristics, the surgeon’s experience, the available surgical resources, and established clinic protocols. However, with no evidence for general superiority, immediate reconstruction might be preferred when it is reasonable to limit the number of surgeries and healthcare costs. Nevertheless, it is imperative that clinical decisions remain under the purview of the treating physician to prevent financial factors from unduly influencing patient care, ensuring alignment with the patient’s needs and desires, including masticatory rehabilitation. In this study, only 18% of patients achieved implant-supported functional masticatory rehabilitation after bony jaw reconstruction. Given the reduced social participation of affected patients, a shift towards expedited, less bureaucratic masticatory rehabilitation using implant-supported dentures is imperative; hence, defining specific implant success criteria for cancer patients is a logical conclusion.

## Figures and Tables

**Table 1:** Comparison of patient demographics, flap type, and treatment characteristics between immediate (n = 72) and delayed (n = 105) reconstruction. DCIA = deep circumflex iliac artery (bone) flap; MFCF = medical femoral condyle flap.

		Immediate Reconstruction (n = 72)		Delayed Reconstruction (n = 105)	
		Mean (min/max)	N (%)	Mean (min/max)	N (%)
Age at reconstruction		59 (14/83)		59 (18/87)	
Gender	male		47 (65.3)		84 (80.0)
	female		25 (34.7)		21 (20.0)
Flap type	fibula flap		55 (76.4)		71 (67.6)
	scapula		9 (12.5)		20 (19.0)
	DCIAF		7 (9.7)		14 (13.3)
	MFCF		1 (1.4)		0
Adjuvant XRT	no		17 (23.6)		16 (15.2)
	yes		55 (76.4)		89 (84.8)
Adjuvant chemotherapy	no		38 (52.8)		48 (45.7)
	yes		34 (47.2)		57 (54.3)
Reconstruction on XRT	no		68 (94.4)		16 (15.2)
	yes		4 (5.6)		89 (84.8)
Local recurrence	no		43 (76.8)		55 (75.3)
	yes		13 (23.2)		18 (24.7)

**Table 2:** Distribution of immediate and delayed reconstruction cases by diagnosis, showing the number and percentage of each type of reconstruction for various conditions, with 72 immediate and 105 delayed reconstruction cases.

Leading Diagnosis	Immediate Reconstruction, N (%)	Delayed Reconstruction, N (%)	Total, N (%)
OSCC	57 (79.2)	76 (72.4)	133 (75.1)
Ameloblastoma	1 (1.4)	4 (3.8)	5 (2.8)
ACC	0	3 (2.9)	3 (1.7)
Giant cell granuloma	1 (1.4)	0	1 (0.6)
Osteosarcoma	0	1 (1.0)	1 (0.6)
Ossifying fibroma	1 (1.4)	1 (1.0)	2 (1.1)
Keratocyst	0	1 (1.0)	1 (0.6)
ORN	8 (11.1)	11 (10.5)	19 (10.7)
Pseudarthrosis	1 (1.4)	0	1 (0.6)
Defect	1 (1.4)	5 (4.8)	6 (3.4)
ARONJ	2 (2.8)	3 (2.9)	5 (2.8)
Total	72 (100)	105 (100)	

**Table 3:** The table presents the flap survival rates in immediate and delayed reconstruction procedures at two universities (University A and University B). The flap survival rates are depicted as a percentage, with chi-square (P) values provided to assess the statistical significance of the observed differences.

		Reconstruction Timing	Flap Survival		Total	Chi-Square (P)
			No	Yes		
University	A	Immediate Reconstruction	6 (11.3%)	47 (88.7%)	53 (100%) (55%)	0.74
		Delayed Reconstruction	8 (18.2%)	36 (81.8%)	44 (100%) (45%)	
University	B	Immediate Reconstruction	5 (26.3%)	14 (73.7%)	19 (100%)	
		Delayed Reconstruction	10 (16.4%)	51 (83.6%)	61 (100%)	
Total			29 (16.4%)	148 (83.6%)	177 (100%)	

## Data Availability

The datasets used and/or analyzed during the current study are available from the corresponding author upon reasonable request.

## Supplementary Materials

The following supporting information can be downloaded at: https://www.mdpi.com/article/10.3390/cancers16050974/s1.
