# Supplementary material for: The Perfect Timing—Immediate versus Delayed Microvascular Reconstruction of the Mandible
Source: Cancers (Basel). 2024 Feb 28;16(5):974. doi: 10.3390/cancers16050974 (PMC10930935; doi:10.3390/cancers16050974)
Supplement: Supplementary file 1 [file cancers-16-00974-s001.zip › cancers-2854972-supplementary.pdf]

Supplementary Materials

OPS-Codes:

1. **5-771** Partial and total resection of a facial skull bone
2. **5-771.00** Alveolar ridge : without reconstruction
3. **5-771.01** Alveolar ridge : reconstruction with non-vascularized graft
4. **5-771.02** Alveolar ridge : reconstruction with free microvascular flap
5. **5-771.03** Alveolar ridge : reconstruction of soft and hard tissue (including alloplastic reconstruction)
6. **5-772** Partial and total resection of the mandible
7. **5-772.2** Hemimandibulectomy
8. **5-772.20** Hemimandibulectomy : without reconstruction
9. **5-772.21** Hemimandibulectomy : reconstruction with non-vascularized graf
10. **5-772.22** Hemimandibulectomy : reconstruction with free microvascular flap
11. **5-772.23** Hemimandibulectomy : alloplastic reconstruction
12. **5-772.2x** Hemimandibulectomy : other
13. **5-772.3** Mandibulectomy, (sub-)total
14. **5-772.30** Mandibulectomy, (sub-)total : without reconstruction
15. **5-772.31** Mandibulectomy, (sub-)total : reconstruction with non-vascularized graft
16. **5-772.32** Mandibulectomy, (sub-)total : reconstruction with free microvascular flap
17. **5-772.33** Mandibulectomy, (sub)total : alloplastic reconstruction
18. **5-772.3x** Mandibulectomy, (sub)total : other
19. **5-772.4** Mandibulectomy, radical (with surrounding tissue)
20. **5-772.40** Mandibulectomy, radical (with surrounding tissue) : without reconstruction
21. **5-772.41** Mandibulectomy, radical (with surrounding tissue) : reconstruction with non-vascularized graft
22. **5-772.42** Mandibulectomy, radical (with surrounding tissue) : reconstruction with free microvascular flap
23. **5-772.43** Mandibulectomy, radical (with surrounding tissue) : alloplastic reconstruction
24. **5-772.4x** Mandibulectomy, radical (with surrounding tissue) : other
25. **5-858.80** Transplantation of an osteomyocutaneous or osteofasciocutaneous flap : head and neck
26. **5-858.30** Harvesting of an osteomyocutaneous or osteofasciocutaneous flap : head and neck.
